# Identification of a Favorable Prognostic Subgroup in Oral Squamous Cell Carcinoma: Characterization of ITGB4/PD-L1^high^ with CD8/PD-1^high^

**DOI:** 10.3390/biom13061014

**Published:** 2023-06-19

**Authors:** Si-Rui Ma, Jian-Feng Liu, Rong Jia, Wei-Wei Deng, Jun Jia

**Affiliations:** 1 The State Key Laboratory Breeding Base of Basic Science of Stomatology (Hubei-MOST) & Key Laboratory of Oral Biomedicine Ministry of Education, School and Hospital of Stomatology, Wuhan University, Wuhan 430079, China; 2 Department of Oral Maxillofacial-Head Neck Oncology, School and Hospital of Stomatology, Wuhan University, Wuhan 430079, China

**Keywords:** ITGB4, oral squamous cell carcinoma, PD-1/PD-L1 axis, CD8, prognosis

## Abstract

Integrin β4 (ITGB4) is a member of the integrin family, which plays a crucial role in mediating cell adhesion to the extracellular matrix. Recent studies have demonstrated that ITGB4 is involved in tumorigenesis and metastasis during the development of cancer. However, the role of ITGB4 in oral squamous cell carcinoma (OSCC) remains unclear. A Multiplex immunohistochemistry (OPAL™, mIHC) assay was employed to stain ITGB4, ALDH1, PD-L1, cytokeratin (CK), CD8 and PD-1 in a human OSCC tissue microarray, containing 26 normal oral epithelium samples, 21 oral epithelium dysplasia samples and 76 OSCC samples. The expression pattern and clinicopathological characteristics of ITGB4 were analyzed and compared with those of PD-1, PD-L1, ALDH1 and CD8. The correlation between subgroups of tumor cells, including ITGB4^+^PD-L1^+^ and ITGB4^+^ALDH1^+^, and subgroups of T cells, including CD8^+^ and CD8^+^PD-1^+^, was evaluated using two-tailed Pearson’s statistics. A Kaplan–Meier curve was built, and a log-rank test was performed to analyze the survival rate of different subgroups. The mIHC staining results show that ITGB4 was mostly expressed in the tumor cells, with a significant increase in the OSCC specimens compared with normal oral epithelium and oral epithelium dysplasia. The paired analysis, conducted between the OSCC tumor tissue and normal paracancer mucosa, confirmed the results. The study further revealed that ITGB4^+^PD-L1^+^ cancer cells, but not ITGB4^+^ALDH1^+^ cancer cells, were significantly associated with the infiltration of CD8^+^ T cells (positivity *p* = 0.005, positive number *p* = 0.03). Additionally, ITGB4^+^PD-L1^+^ tumor cells were positively correlated with CD8^+^PD-1^+^ T cells (positivity *p* = 0.02, positive number *p* = 0.03). Most intriguingly, the subgroup of ITGB4/PD-L1^high^ with CD8/PD-1^high^ displayed the best prognosis compared with the other considered subgroups. The results show that the expression of ITGB4 was increased in OSCC compared with normal oral mucosa. Furthermore, a specific subgroup with high levels of expression of ITGB4/PD-L1 and CD8/PD-1 was found to have a relatively better prognosis compared with the other subgroups. Ultimately, this study sheds light on the potential role of ITGB4 in OSCC and provides a basis for further investigation.

## 1. Introduction

Oral squamous cell carcinoma (OSCC), which displays increasing numbers of diagnosed cases and deaths annually, is a variety of heterogeneous malignancy that arises from the epithelia of oral cavities [1]. Depending on the disease stage, advanced OSCC is generally treated with surgical resection, followed by radiation or chemotherapy plus radiation (chemoradiation, CRT) [2]. Regardless of the benefits of the primary treatment options developed over the past several decades, they are frequently ineffective and cause no significant improvement in the prognosis of OSCC patients [3].

Integrins are a group of heterodimeric transmembrane receptor manipulating the interaction of cells with extracellular matrix components [4]. Integrin β4 (ITGB4), which heterodimerizes exclusively with the α6 chain, functions as a receptor for the basement membrane protein laminin. It influences cellular structure, cell cycle progression, differentiation and survival [5]. Aside from its pivotal functions in embryonic development and tissue organization, in vitro and in vivo experiments have indicated that ITGB4 promotes tumorigenesis and metastasis in a broad range of solid tumors, including breast cancer, prostate cancer, lung cancer and pancreatic cancer, by triggering downstream signaling. It performs this primarily through oncogenic receptor tyrosine kinases, including ErbB2, PI3K, FAK/AKT and c-Met [6,7,8,9]. Furthermore, ITGB4 has been identified as a cancer stem cell (CSC) marker that operates by promoting metastasis, self-renewal, tumor propagation and chemotherapy resistance in triple-negative breast cancer and non-small-cell lung cancer [10,11,12,13]. ITGB4 expression patterns display a strong heterogeneity and may vary between different carcinomas [14,15,16,17,18]. Hence, the prognostic value of ITGB4 is distinct. Historically, on the basis of analyses in human cancer tissue samples, ITGB4 is regarded as an unfavorable prognosis marker in breast cancer, lung cancer and colorectal cancer [19,20,21]. Instead, it has since been found that a high level of ITGB4 indicates a prolonged survival rate in esophageal squamous cell carcinoma [22,23]. Moreover, it has been reported that the loss or dissociation of ITGB4, which leads to the breakup of the cell basement membrane, could be related to an increased risk of metastasis in prostate cancer [23,24]. Although the array of published studies has revealed the potential diagnostic and prognostic value of ITGB4 in large varieties of cancer, accurate expression patterns and prognoses of ITGB4 in OSCC are still incompletely understood.

For the last decades, a deep understanding of the role of the immune system in cancer progression has provided knowledge of the mechanisms behind cancer immunosurveillance evasion. The discovery and development of specific immune checkpoints have demonstrated therapeutic potential in cancer treatment. Researchers have found evidence that hampered immunogenicity is associated with elevated immune dysfunction in OSCC, suggesting a negative impact on the prognosis [2]. Immune checkpoint inhibitors such as nivolumab and pembrolizumab, which target the PD-1/PD-L1 axis, have been approved by the FDA for the management of patients with recurrent or metastatic head and neck squamous cell carcinoma (HNSCC) [25,26,27]. Aside from the important role it plays in cancer immunotherapy, the PD-1/PD-L1 axis is also regarded as a potential prognostic indicator. Although studies on the prognosis of the PD-1/PD-L1 axis have emerged one after another, their conclusions remain controversial. For example, in nasopharyngeal carcinoma, a higher level of PD-1 positive infiltrated immune cells indicates a longer survival rate [28]. However, in salivary gland carcinomas, the opposite conclusion of the prognosis judgment based on the expression of PD-1 was observed [29]. As with PD-1, the outcome predictive role of PD-L1 expression is still controversial. In OSCC, PD-L1 expression in more than 10% of tumor cells is closely related to disease recurrence and lower disease-specific survival. Instead, another study reported that high PD-L1 expression in OSCC is not significantly related to overall survival rate [30]. Considering the discrepancy between these published conclusions and the complexity of the tumor immune microenvironment, we suggest that the use of novel approaches such as multi-biomarker analysis may furnish us with a better understanding of the prognostic value of the PD-1/PD-L1 axis.

In this study, we explored the expression pattern and clinicopathological characteristics of ITGB4 by mIHC. The relationship among ITGB4, PD-1, PD-L1, ALDH1 and CD8 was evaluated. In addition, we considered that combined high expression levels of ITGB4, PD-L1, PD-1 and CD8 in OSCC patients may act as a novel immunophenotyping system that predicts the favorability of a prognosis.

## 2. Materials and Methods

### 2.1. The Human OSCC Samples

Human OSCC tissues were obtained from the surgical specimens of OSCC patients in the department of Oral and Maxillofacial-head neck oncology, Stomatology of Wuhan University. All patients were pathologically diagnosed with OSCC and received radical surgery. Informed consent was provided by all patients prior to surgery.

### 2.2. The Human OSCC Tissue Microarray

The human OSCC tissue microarray used in this study contained 26 normal oral epithelia, 21 oral epithelial dysplasia and 76 OSCC. Of the 76 primary OSCC patients, we followed up with 60 patients until the end of the study or death, and we could not follow up with 16 patients. The clinical and pathological parameters of the 60 primary oral squamous cell carcinomas with follow-up in this study are shown in Supplementary Table S1. The tissue microarray contained 15 paired OSCC tumor tissues and normal paracancer mucosa.

### 2.3. The Multiplex Immunohistochemistry Staining

The multiplex immunohistochemistry staining was conducted using the Opal 7-Color Manual IHC Kit (NEL811001KT, PerkinElmer, Waltham, MA, USA) according to the operation instructions. In brief, deparaffinization and rehydration were performed through xylenes and graded alcohol. Then, the antigen retrieval was completed in an AR6 buffer in a microwave oven. Endogenous peroxidase was blocked with hydrogen peroxide (3%), and non-specific binding was blocked with goat serum. The slide was incubated with the primary antibody overnight at 4 ℃. On the next day, the slide was incubated with an HRP labelled secondary antibody. After being washed, the slide was incubated with tyramide signal amplification (TSA, PerkinElmer Opal kit, including Opal 520, Opal 540, Opal 570, Opal 620, Opal 650, and Opal 690). After that, stripping was performed in a microwave oven with 1 × AR6 buffer. Additionally, the staining cycles were repeated until all the primary antibodies (ITGB4, PD-L1, ALDH1, CD8, PD-1 and CK) were incubated. Following the completion of all procedures, tissue sections were incubated with DAPI. The primary antibody used in this study contained the following: ITGB4 (Cat: 14803; CST); PD-L1 (Cat: 13684; CST); ALDH1 (Cat: 54135; CST); CD8 (Cat: 85336; CST); PD-1 (Cat: 86163; CST); and CK (Cat: 0671, MXB biotechnologies).

### 2.4. Quantification Analysis

The slide was scanned with the PerkinElmer Vectra (2.0.8), and the images were prepared and analyzed with the InForm Advanced Image Analysis Software (2.0). The epithelium area and stroma area were distinguished via CK staining. The positivity and positive number of the makers were calculated in the tumor area and stroma area, respectively.

### 2.5. The Survival Analysis

In order to perform the survival analysis, the Kaplan–Meier curve and log-rank test were used. The median of the positivity or positive number was selected as the cut-off. To further explore the prognostic value of the subgroup, the cohort was divided into four parts according to the ITGB4^+^PD-L1^+^ cancer cells and CD8^+^PD-1^+^ T cells. Then, the survival curve of each subgroup was compared. The survival of ITGB4 was also analyzed using the free available Pan cancer database (GEPIA, http://gepia.cancer-pku.cn/, assessed on 23 May 2023).

### 2.6. The Statistical Analysis

The differences among groups were analyzed using a one-way ANOVA followed by the post-Tukey test. A paired *t*-test or unpaired *t*-test was to used evaluate the differences between two groups. The correlation analysis was performed using two-tailed Pearson’s statistics. The statistical significance was identified as *p* < 0.05. All analyses were performed using GraphPad Prism 8.0 (Graph Pad Software Inc., CA, USA).

## 3. Results

### 3.1. Multiplex Immunohistochemistry Staining of ITGB4, ALDH1, PD-L1, CD8 and PD-1 in OSCC Specimens

**Figure 1:**
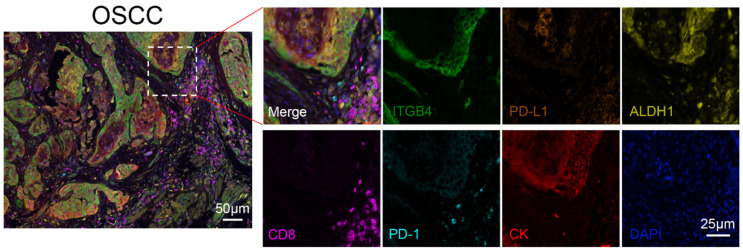
Representative image of multiplex immunohistochemistry (mIHC) for the specimen stained with ITGB4, ALDH1, PD-L1, CD8, PD-1 and cytokeratin (CK). ITGB4: green; PD-L1: orange; ALDH-1: yellow; CD8: magenta; PD-1: cyan; CK: red; DAPI: blue. Scale bar = 25 μm.

First, the expression of ITGB4 was evaluated with multiplex immunohistochemistry (OPAL™, mIHC) in the OSCC tissue microarray. Second, the positive proportions and positive numbers of cancer stem cells (ALDH1^+^ cancer cells), inhibitory immune checkpoints on cancer cells (PD-L1^+^ cancer cells) and immune cell subset (CD8^+^ T cells, PD-1^+^ cells) were analyzed. As shown in Figure 1, the fluorescence of Opal 690 (red) was stained with cytokeratin (CK) in order to identify the area of the epithelia, with the remaining area regarded as tumor stroma. The fluorescence of Opal 520 (green) indicated the population of ITGB4^+^ cells, which was predominantly expressed in tumor cells. The fluorescence of Opal 570 (yellow) represented the population of ALDH1^+^ cells, and the fluorescence of Opal 620 (orange) displayed the population of PD-L1^+^ cells. Both populations were mostly located in tumor cells, with some examples present in the stroma. The fluorescence of Opal 650 (magenta) showed the population of CD8^+^ cells, which were expressed in the area of the tumor stroma. The fluorescence of Opal 540 (cyan) demonstrated the population of PD-1^+^ cells, which were also mainly expressed in the area of the tumor stroma.

### 3.2. The Expression of ITGB4 Is Dramatically Increased in OSCC Specimen

**Figure 2:**
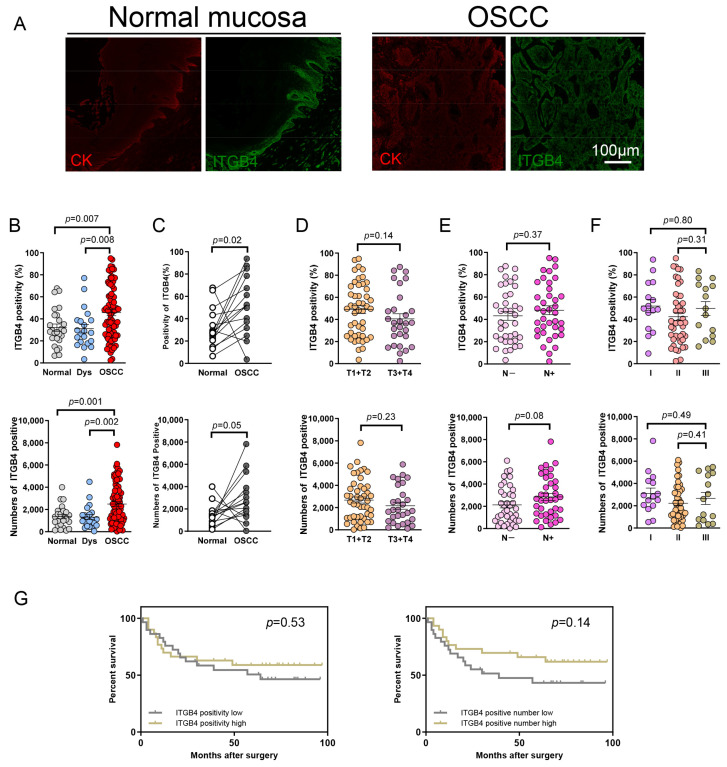
Expression of ITGB4 was dramatically increased in OSCC specimen. (**A**) Image of ITGB4 (green) and CK (red) in normal mucosa and OSCC specimen. Scale bar = 100 μm. (**B**) Statistical results of ITGB4 positivity and ITGB4 positive number in normal mucosa (Normal), dysplasia (Dys) and OSCC samples. (**C**) Paired *t*-test analysis between OSCC sample and normal paracancer mucosa. The quantitative analysis of ITGB4 expression was performed for tumor size ((**D**) T1 + T2 vs. T3 + T4), lymph node status ((**E**), N− vs. N+) and pathological grade ((**F**), I vs. III, II vs. III). (**G**) Kaplan–Meier curve of overall survival of OSCC patients with high expression of ITGB4 and low expression of ITGB4 (positivity: left; positive number: right).

Then, we evaluated the expression of ITGB4 in OSCC specimens. As shown in Figure 2A, ITGB4 staining occurred in the tumor cells (mostly in the invasion frontier) and the basal layer of the normal oral mucosa. The results of quantitative analysis revealed that positive proportions and positive numbers of ITGB4 in tumor samples were significantly higher than the expression seen in normal mucosa (positivity *p* = 0.007, positive number *p* = 0.001, Figure 2B) and dysplasia (positivity *p* = 0.008, positive number *p* = 0.002, Figure 2B). In order to confirm these results, the paired OSCC tumor tissue and normal paracancer mucosa were selected for further analysis. As shown in Figure 2C, the paired *t*-test demonstrated that the expression of ITGB4 was distinctly higher in OSCC samples, as compared with normal paracancer mucosa (positivity *p* = 0.02, positive number *p* = 0.05). The correlation was explored between the expression of ITGB4 and clinical–pathological parameters. The results showed that there was no significant difference between the T1 + T2 and T3 + T4 stages (Figure 2D). In the lymph node status, although the expression of ITGB4 was also not significantly different between the positive status and negative status, the positive numbers of ITGB4 indicated an increasing tendency (*p* = 0.08, Figure 2E). In addition, the analysis of the relationship between the expression of ITGB4 and pathological grades did not reach a statistically significant difference (Figure 2F).

The potential prognostic value of ITGB4 expression in OSCC patients was further explored. The median value of ITGB4 positivity and positive numbers were selected as the cut-off. The result from the Kaplan–Meier curve revealed that neither ITGB4 positivity nor ITGB4 positive numbers were correlated with the prognosis (Figure 2G). The survival analysis based on the GEPIA also did not reach significance (Supplementary Figure S1).

Based on the mIHC, the subtype of the double-positive cells was analyzed in this study. As depicted in Supplementary Figure S2A,B, it seems that the proportion and cell numbers of double-positive ITGB4 and PD-L1 cancer cells (ITGB4^+^PD-L1^+^) was high in the OSCC specimen, and the statistical result approached a significant difference (positivity *p* = 0.07, positive number *p* = 0.06). This may be attributed to the limited numbers of the sample size. In addition, the subtype of the double-positive of the ITGB4 and ALDH1 (ITGB4^+^ALDH^+^) expression and CD8^+^PD-1^+^ T cells were not significantly increased in OSCC samples.

### 3.3. ITGB4^+^PD-L1^+^ Cancer Cells Are Significantly Associated with Infiltration of CD8^+^ T Cells

**Figure 3:**
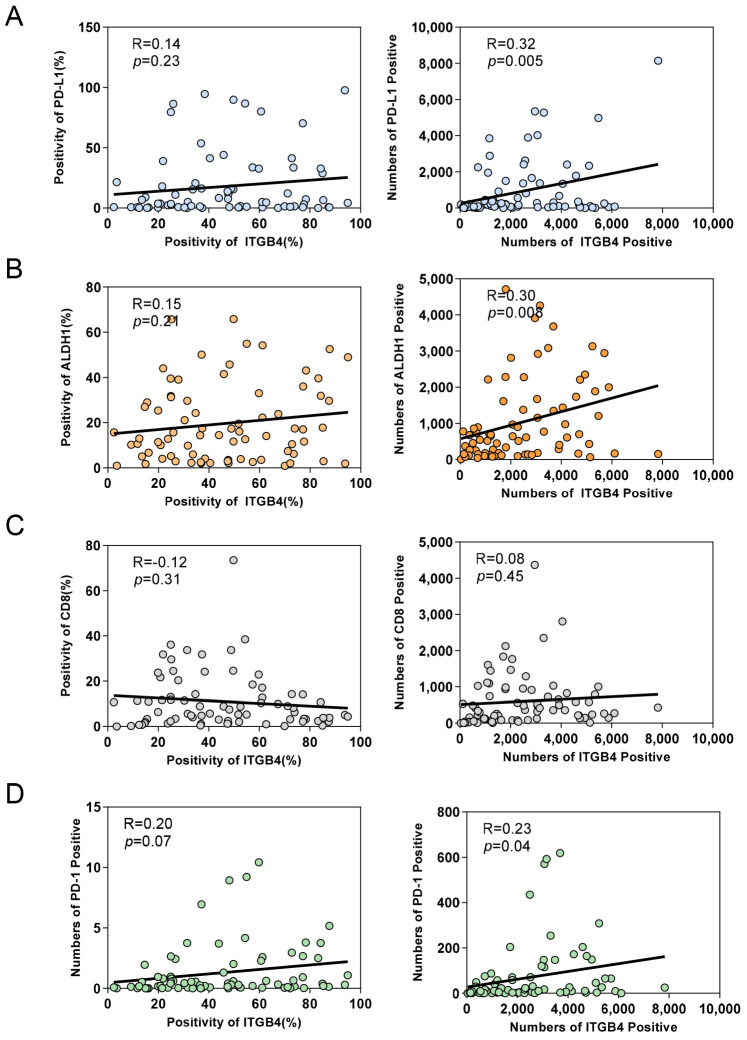
Correlation analysis based on mIHC. The Spearman correlation coefficient test showed the relationship between ITGB4 with PD-L1 ((**A**) positivity *p* = 0.23, positive number *p* = 0.005), ALDH1 ((**B**), positivity *p* = 0.21, positive number *p* = 0.008), CD8 ((**C**), positivity *p* = 0.31, positive number *p* = 0.45) and PD-1 ((**D**), positivity *p* = 0.07, positive number *p* = 0.04) in OSCC samples.

To investigate the relationship between ITGB4 and the above-mentioned molecules, Pearson’s correlation coefficient test was used to analyze the correlation between ITGB4 and PD-L1, ALDH1, CD8 and PD-1. In the tumor area, the results showed a statistically significant association between ITGB4 and PD-L1 in terms of positive numbers (*p* = 0.005), but not in terms of positivity (*p* = 0.23, Figure 3A). Similarly, ITGB4 was found to have a meaningful relationship with ALDH1 in terms of positive numbers (*p* = 0.008), but not in terms of positivity (*p* = 0.21, Figure 3B). Moreover, no significant relationship was found between ITGB4 and CD8 (Figure 3C). However, the expression of ITGB4 was significantly associated with PD-1 (positivity *p* = 0.07, positive number *p* = 0.04, Figure 3D).

**Figure 4:**
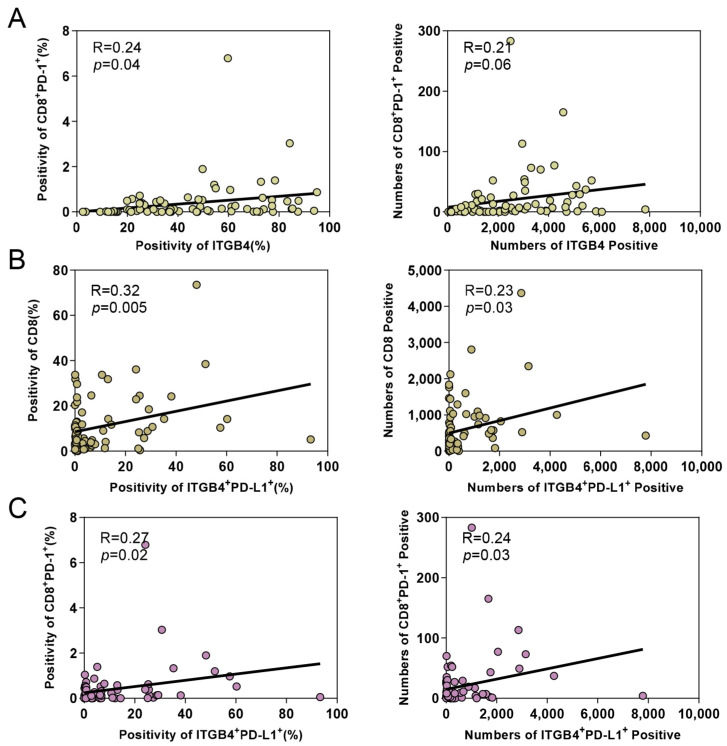
ITGB4^+^PD-L1^+^ cancer cells were significantly associated with infiltration of CD8^+^ T cells. (**A**) Spearman correlation coefficient test displayed the association between ITGB4 cancer cells and CD8^+^PD-1^+^ exhausted T cells in OSCC specimens (positivity *p* = 0.04, positive number *p* = 0.06). (**B**) Correlation analysis between ITGB4^+^PD-L1^+^ cancer cells and CD8^+^ T cells (positivity *p* = 0.005, positive number *p* = 0.03). (**C**) Correlation analysis between ITGB4^+^PD-L1^+^ cancer cells and CD8^+^PD-1^+^ exhausted T cells (positivity *p* = 0.02, positive number *p* = 0.03).

The mIHC provided precise cell colocalization and an appropriate correlation between cells. In the case of tumors, CD8^+^PD-1^+^ cells were identified as exhausted T cells. Of note, the results show that ITGB4 was statistically associated with CD8^+^PD-1^+^ cells (positivity *p* = 0.04, positive number *p* = 0.06, Figure 4A). ITGB4^+^PD-L1^+^ tumor cells were found to be significantly associated with CD8^+^ T cells (positivity *p* = 0.005, positive number *p* = 0.03, Figure 4B). In contrast, ITGB4^+^ALDH1^+^ cancer cells were not correlated with CD8 (Supplementary Figure S3A). More interestingly, when the relationship between ITGB4^+^PD-L1^+^ tumor cells and CD8^+^PD-1^+^ T cells was analyzed, their correlation was found to be statistically different (positivity *p* = 0.02, positive number *p* = 0.03, Figure 4C). Overall, these results demonstrate that ITGB4^+^PD-L1^+^ tumor cells may induce the accumulation of CD8^+^ T cells and consequently result in the exhaustion of CD8^+^ T cells.

### 3.4. The Subgroup of ITGB4/PD-L1^high^ with CD8/PD-1^high^ Indicated a Relatively Superior Prognosis Compared to Other Subgroups

**Figure 5:**
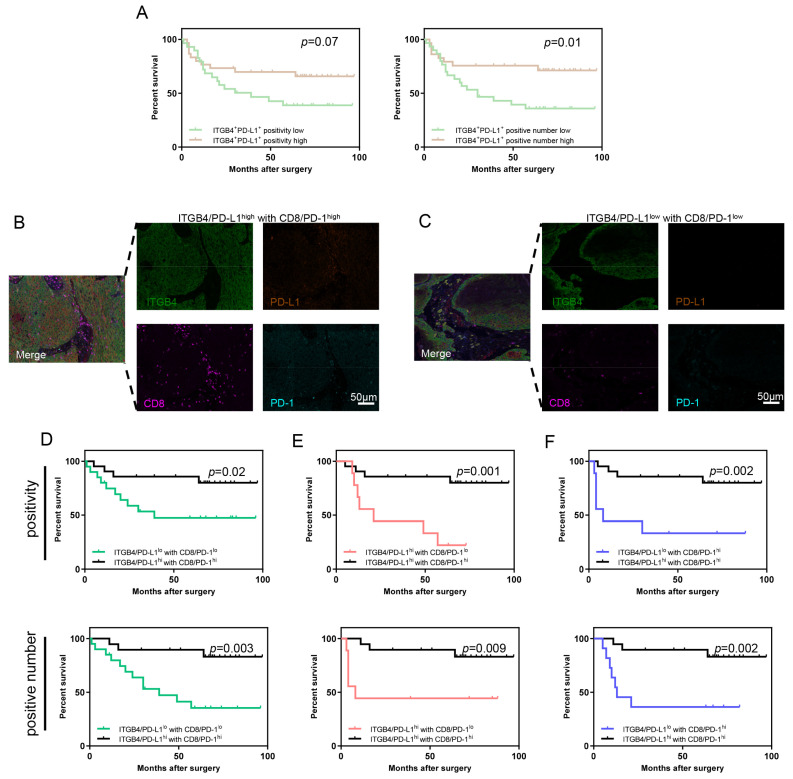
The subgroup of ITGB4/PD-L1^high^ with CD8/PD-1^high^ indicated the relative best prognosis compared with other subgroups. (**A**) Kaplan–Meier curve of overall survival of OSCC patients with high ITGB4^+^PD-L1^+^ cancer cells and low ITGB4^+^PD-L1^+^ cancer cells. (left: positivity *p* = 0.07; right: positive number *p* = 0.01). (**B**) Representative images of ITGB4/PD-L1^high^ with CD8/PD-1^high^. (**C**) Representative images of ITGB4/PD-L1^low^ with CD8/PD-1^low^. (**D**) Kaplan–Meier curve of overall survival of OSCC patients between ITGB4/PD-L1^high^ with CD8/PD-1^high^ and ITGB4/PD-L1^low^ with CD8/PD-1^low^ (upper: positivity *p* = 0.02, lower: positive number *p* = 0.003). (**E**) Kaplan–Meier curve of overall survival of OSCC patients between ITGB4/PD-L1^high^ with CD8/PD-1^high^ and ITGB4/PD-L1^high^ with CD8/PD-1^low^ (upper: positivity *p* = 0.001, lower: positive number *p* = 0.009). (**F**) Kaplan–Meier curve of overall survival of OSCC patients between ITGB4/PD-L1^high^ with CD8/PD-1^high^ and ITGB4/PD-L1^low^ with CD8/PD-1^high^ (upper: positivity *p* = 0.002, lower: positive number *p* = 0.002).

The prognostic differences between patients with ITGB4/PD-L1^low^ and patients with ITGB4/PD-L1^high^ were compared. The results show that patients with ITGB4/PD-L1^high^ had a better survival rate compared to those with ITGB4/PD-L1^low^ (positivity *p* = 0.07, positive number *p* = 0.01, Figure 5A). In contrast, no differences were observed between patients with ITGB4/ALDH1^low^ and ITGB4/ALDH1^high^ (Supplementary Figure S3B). Given the close relationship between ITGB4/PD-L1 tumor cells and CD8/PD-1 T cells, the patients were divided into four subgroups based on the expression of these cells. These subgroups were the following: ITGB4/PD-L1^high^ with CD8/PD-1^high^, ITGB4/PD-L1^high^ with CD8/PD-1^low^, ITGB4/PD-L1^low^ with CD8/PD-1^high^ and ITGB4/PD-L1^low^ with CD8/PD-1^low^. The representative images of ITGB4/PD-L1^high^ with CD8/PD-1^high^ and ITGB4/PD-L1^low^ with CD8/PD-1^low^ are displayed in Figure 5B,C. After comparing the survival rates of these subgroups in pairs, an interesting outcome was observed. The subgroup of ITGB4/PD-L1^high^ with CD8/PD-1^high^ had the best survival rate compared to ITGB4/PD-L1^low^ with CD8/PD-1^low^ (positivity *p* = 0.02, positive number *p* = 0.003, Figure 5D), ITGB4/PD-L1^high^ with CD8/PD-1^low^ (positivity *p* = 0.001, positive number *p* = 0.009, Figure 5E) and ITGB4/PD-L1^low^ with CD8/PD-1^high^ (positivity *p* = 0.002, positive number *p* = 0.002, Figure 5F). This conclusion was based on an assessment of both positivity and positive numbers. Additionally, with the exception of the subgroup of ITGB4/PD-L1^high^ with CD8/PD-1^high^, no significant differences were found among the other subgroups (Supplementary Figure S4A–C). These findings suggest that the specific subgroup of ITGB4/PD-L1^high^ with CD8/PD-1^high^ had the best prognosis.

## 4. Discussion

Prior to conducting our work, evidence had begun to accumulate evidence that indicated that ITGB4 exhibits an aberrant expression and frequently correlates with malignant progression in a board range of carcinomas [6,10]. Additionally, ITGB4 has been linked to the clinicopathological characteristics of cancer, such as tumor stage and pathological grade [31,32]. In accordance with these published studies, we found ITGB4 expression to be significantly elevated in postoperative OSCC tissues when compared with normal oral mucosa or dysplasia. These findings indicate the potential oncogenic role of ITGB4 in OSCC. However, when evaluating the association of ITGB4 with clinicopathological characteristics in OSCC, we found that the ITGB4 expression level is not significantly related to tumor size, lymph node metastasis or pathological grade. These discrepancies potentially resulted from the limited number of considered cases in the present study. Despite the fact that the conclusion regarding the prognostic value of ITGB4 is controversial in different studies on different cancers, we found a high level of ITGB4 to be associated with a relatively prolonged survival in OSCC patients, even though the results were not statistically significant. A comprehensive study on esophagus squamous cell carcinoma distinguished the expression pattern of ITGB4 into pathological and physiological states. Briefly, a physiological state means that the expression of ITGB4 was confined to the basal layer of neoplastic cells adjacent to the tumor stroma. Conversely, a pathological expression means that ITGB4 was found throughout the tumor tissues [22]. Furthermore, several studies have reported that the expression of ITGB4 throughout the tumor tissue correlates with a poor prognosis [33,34]. Instead, abrogation or the loss of ITGB4 basal layer expression in cancers is related to the level of malignancy in SCC [33,35,36]. Based on the above studies, we hypothesized that the favorable trend in the prognosis of OSCC patients in this current study may be related to the high ITGB4 level in the tumor basal layer. It was evident that further comprehensive studies into the distinct expression state of ITGB4 and its effect on prognosis in OSCC are required.

In order to investigate the potential oncogenic functions of ITGB4 in OSCC, we analyzed the correlation among the expression of ITGB4, ALDH1, PD-L1, CD8 and PD-1, finding the ITGB4 level to be significantly correlated with ALDH1, PD-L1 and PD-1. In multiple malignance types, cancer stem cells (CSCs), a group of highly tumorigenic, self-renewing tumor cell sub-population that is resistant to traditional radiation and chemotherapy, could be enriched by virtue of their increased expression of aldehyde dehydrogenase (ALDH) activity, as accessed by the Aldefluor assay [37,38]. In prostate cancer, the silencing of ITGB4 reduces the ability of tumor progenitor cells to self-renew in vitro, suggesting that ITGB4 signaling may play a critical role in maintaining CSCs within the tumor [39]. Additionally, a similar effect was observed in triple-negative breast cancer [10]. The present study demonstrates that ITGB4 expression in OSCC was statistically associated with ALDH1, a biomarker of CSCs, indicating the potential relevance of ITGB4 and CSCs. Further investigation into the functional and regulatory role of ITGB4 in CSCs is needed. OSCC tissues, for instance, are in an immunosuppressive state [40]. Immune checkpoints (ICs) are immunosuppressive molecules with the capacity to maintain self-tolerance by modulating T cell function and protecting surrounding tissues by suppressing immune responses. In addition to traditional treatments, targeting immune checkpoints has become another therapeutic option for OSCC [25,26]. According to our observations, we found ITGB4 expression to be statistically correlated with PD-1 and PD-L1 expression in OSCC tissue, indicating the potential immunogenicity of ITGB4 in OSCC. Comprehensive functional and mechanical studies of ITGB4 in the immuno-oncology of OSCC are needed in the future.

Over the past decades, the impact of the tumor immune microenvironment (TME) on the prognosis of cancer patients has received increasing attention [41]. It has long been known that the presence of intra-tumoral T cells is correlated with better outcomes in a number of human malignancies. For example, it has been appreciated for over 20 years that the presence of brisk T cell infiltrates is associated with improved overall survival in melanoma [42]. Moreover, the magnitude of tumor infiltrated T cells was shown to form an independent favorable prognostic indicator in colorectal cancer [43]. PD-1 is an inhibitor of both adaptive and innate immune responses and is expressed in exhausted T cells. Of note, PD-1 was reported as being highly expressed in tumor-specific T cells [44]. In the present study, we found ITGB4 expression levels to be significantly associated with CD8^+^PD-1^+^ T cells. Hence, we speculate that the recruitment and infiltration of CD8^+^ cells may be due to the interaction of immune cells with ITGB4^+^ tumor cells at the tumor– stroma margin. However, these CD8^+^ cells were consequently exhausted by the immunosuppressive tumor microenvironment. Following T cell activation, pro-inflammatory cytokines, such as interferon γ (IFN-γ), were secreted to exert an anti-tumoral effect. IFN-γ is a pro-inflammatory cytokine produced by T cells and NK cells that enhances the major histocompatibility complex (MHC) expression to promote neoantigen presentation in tumor cells by harnessing the IFN-γ/JAK/STAT1 pathway, thus expressing PD-L1 in cancer cells, inactivating T cells and attenuating immunosurveillance in the TME [45]. Although PD-L1 expression in cancer has been defined as a prognostic factor not only in patients receiving conventional treatments, but also in patients receiving immunotherapy targeting PD-1 or PD-L1 [42], previous studies have reported conflicting conclusions about the prognostic value of PD-L1 expression in OSCC, even in some meta-analyses, including large quantities of data [43,44,45]. Considering the discordance, we attempted to perform multi-biomarker analyses to obtain more comprehensive results. In OSCC tissues, the co-expression of ITGB4 and PD-L1 was remarkedly related to not only CD8^+^ T cells, but also CD8^+^PD-1^+^ exhausted T cells. We speculate that the augmented infiltrated exhausted T cells in high ITGB4-expressing tumor cells induced a sequencing elevated level of PD-L1. Moreover, with a synergic analysis of ITGB4 and PD-L1 expression levels, we found that high levels of ITGB4 and PD-L1 in the tumor tissues of OSCC patients indicate a remarkably favorable prognosis. Furthermore, considering the positive prognostic role of activated T cells in cancers, we explored a subgroup of patients with a high co-expression of ITGB4, PD-L1, CD8 and PD-1. The results show that this subgroup of OSCC patients had a significantly favorable prognosis when compared with any other subgroup. Nevertheless, the underlying mechanisms involved in this phenomenon require further study.

The results comprehensively describe alterations in both the expression and prognostic value of ITGB4 in postoperative OSCC tissues, which strongly encourages further investigation. Despite ITGB4 being correlated to CSCs, it seemed to act as a positive outcome predicator when synergically analyzed with the PD-1/PD-L1 axis as well as CD8. Subsequent studies with a larger scale of cases incorporated with in vitro and in vivo mechanical studies can extend our understanding of ITGB4 in the progression of OSCC.

## 5. Conclusions

In this study, via the practice of novel mIHC staining on a human OSCC tissue microarray, we explored ITGB4 expression patterns and the relationships between ITGB4 expression levels and clinicopathological characteristics. Additionally, the correlation among ITGB4, PD-1, PD-L1, ALDH1 and CD8 in human OSCC tissues were investigated. Notably, we observed a remarkable prognostic benefit in patients with high levels of co-expressed ITGB4, PD-L1, CD8 and PD-1 (ITGB4^high^PD-L1^high^ with CD8^high^PD-1^high^), indicating that ITGB4, PD-L1, CD8 and PD-1 can be regarded as comprising a potential immunophenotyping system that may act as a prognosis predictive biomarker.

## Data Availability

The data presented in this study are available on request from the corresponding author.

## Supplementary Materials

The following supporting information can be downloaded at https://www.mdpi.com/article/10.3390/biom13061014/s1: Figure S1: The survival curve of ITGB4 in GEPIA database. Figure S2: Intensity analysis of double positive cells in OSCC. Figure S3: Relationship and prognosis analysis of PD104^+^ALDH1^+^ cancer cells. Figure S4: Survival analysis among other subgroups of OSCC patients. Table S1: Clinicopathological features of 60 primary oral squamous cell carcinomas with follow-up in this study.
