# Supplementary material for: Identification of a Favorable Prognostic Subgroup in Oral Squamous Cell Carcinoma: Characterization of ITGB4/PD-L1high with CD8/PD-1high
Source: Biomolecules. 2023 Jun 19;13(6):1014. doi: 10.3390/biom13061014 (PMC10296360; doi:10.3390/biom13061014)
Supplement: Supplementary file 1 [file biomolecules-13-01014-s001.zip › biomolecules-2302475-supplementary.pdf]

Supplementary Figures

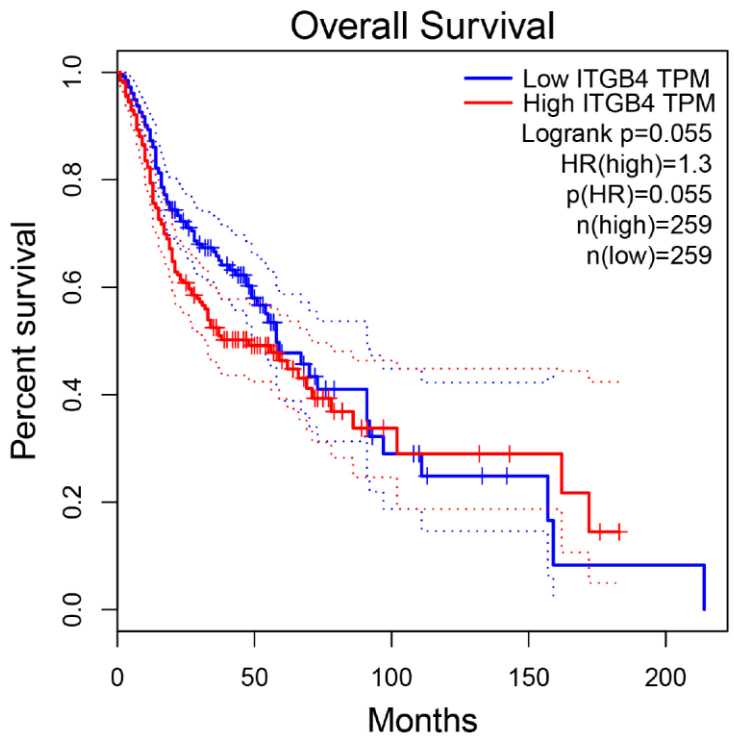

Supplementary Figure S1
The survival curve of ITGB4 in GEPIA database.

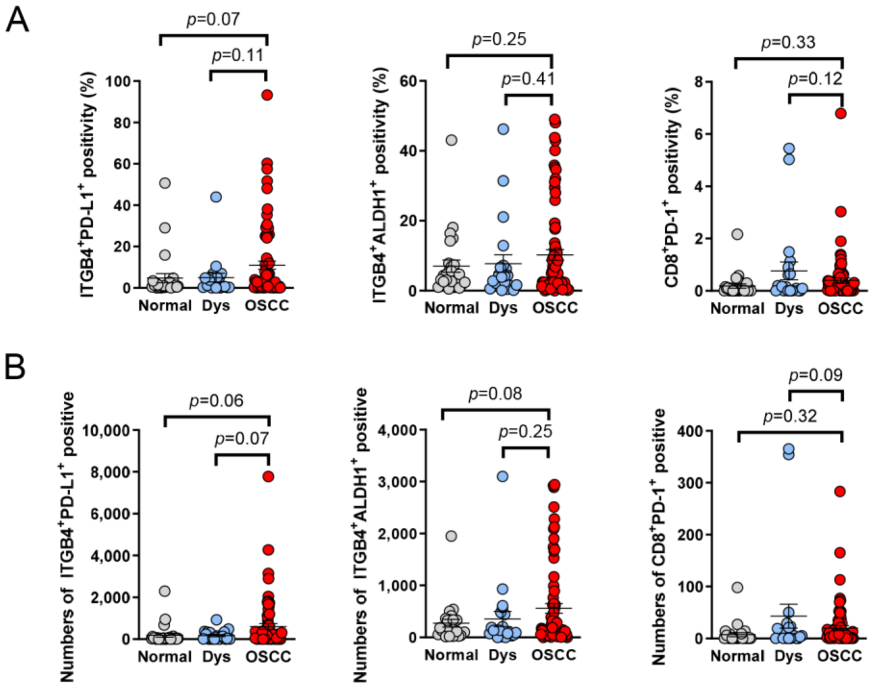

Supplementary Figure S2

The intensity analysis of double positive cells in OSCC. The positivity (A) and positive numbers (B) of ITGB4^{+}PD-L1^{+} cancer cells, ITGB4^{+}ALDH1^{+} cancer cells and CD8^{+}PD-1^{+} T cells in OSCC specimens as compared with normal mucosa and dysplasia based on the multiplex immunohistochemistry (mIHC).

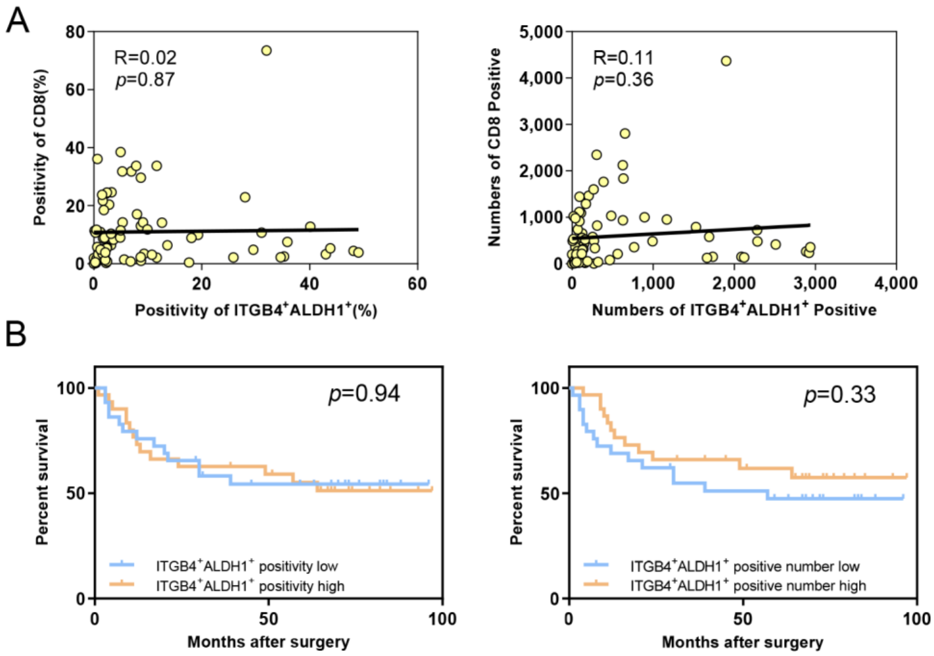

Supplementary Figure S3

The relationship and prognosis analysis of PD104^{+}ALDH1^{+} cancer cells.

A The correlation analysis between PD104^{+}ALDH1^{+} cancer cells and CD8^{+} T cells (positivity p=0.87, positive number p=0.36). B The Kaplan-Meier curve of overall survival of OSCC patients with high ITGB4^{+}ALDH1^{+} cancer cells and low ITGB4^{+}ALDH1^{+} cancer cells. (left: positivity p=0.94; right: positive number p=0.33).

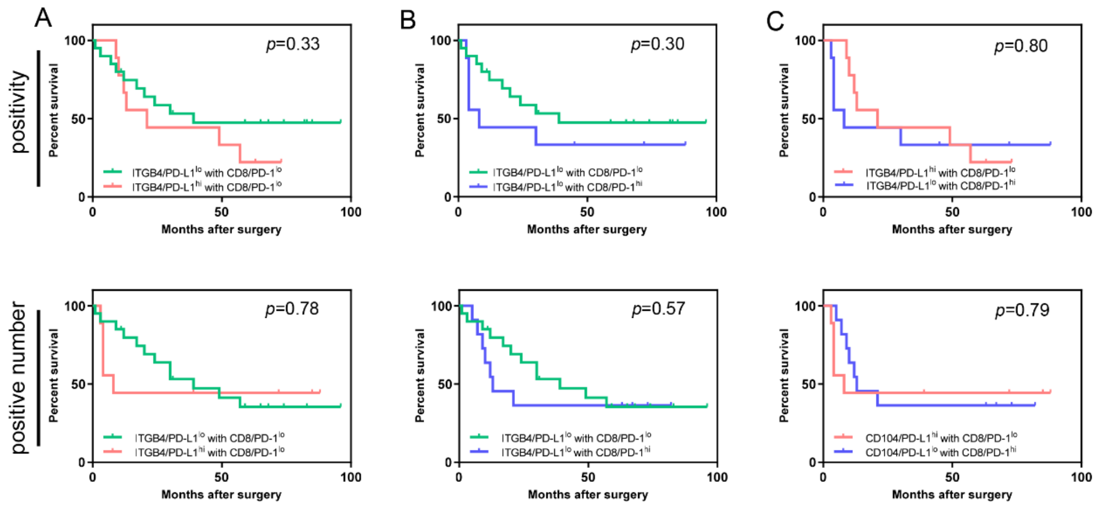

Supplementary Figure S4

The survival analysis among other subgroups of OSCC patients. The Kaplan-Meier curve of overall survival of OSCC patients between (A)ITGB4/PD-L1^{high} with CD8/PD-1^{low} and ITGB4/PD-L1^{low} with CD8/PD-1^{low} (upper: positivity p=0.33, lower: positive number p=0.78), (B) ITGB4/PD-L1^{low} with CD8/PD-1^{high} and ITGB4/PD-L1^{low} with CD8/PD-1^{low} (upper: positivity p=0.30, lower: positive number p=0.57), (C) ITGB4/PD-L1^{low} with CD8/PD-1^{high} and ITGB4/PD-L1^{high} with CD8/PD-1^{low} (upper: positivity p=0.80, lower: positive number p=0.79).

Supplementary Table S1. Clinicopathological features of 60 primary oral squamous cell carcinomas with follow-up in this study.

No.	Gender	Age	TNM stage	Grade	Alive (0) or Dead (1)	Survival time (months)
1	male	49	T2N0M0	I	0	74
2	male	50	T2N1M0	III	1	49
3	male	50	T3N1M0	II	1	9
4	female	43	T2N0M0	II	0	72
5	female	65	T3N0M0	II	1	13
6	female	73	T3N0M0	II	0	68
7	male	40	T3N2M0	II	1	4
8	male	38	T2N1M0	I	0	31
9	male	44	T1N0M0	II	0	59
10	female	67	T2N0M0	II	0	51
11	male	73	T2N1M0	I	0	45
12	male	61	T1N0M0	II	1	39
13	male	68	T2N0M0	II	0	39
14	female	57	T1N0M0	II	0	65
15	male	60	T3N0M0	III	1	3
16	male	40	T2N1M0	II	0	97
17	male	39	T2N0M0	II	0	96
18	male	77	T1N0M0	III	1	5
19	male	68	T2N2M0	I	0	93
20	male	63	T3N1M0	I	1	20
21	male	43	T2N0M0	III	1	30
22	female	78	T4N2M0	II	0	88
23	male	57	T3N1M0	II	1	0
24	male	72	T4N0M0	II	0	85
25	male	62	T4N1M0	II	1	24
26	male	80	T4N0M0	II	1	12
27	male	70	T4N1M0	II	0	84
28	male	72	T2N2M0	II	0	29
29	male	57	T3N1M0	II	0	83
30	male	53	T3N1M0	IIII	1	12
31	male	55	T3N0M0	II	1	8
32	female	66	T1N1M0	III	1	16
33	male	62	T1N0M0	II	0	82
34	male	46	T4N0M0	II	0	82
35	male	54	T4N1M0	I	1	11
36	male	54	T2N1M0	I	0	79
37	male	41	T2N1M0	III	0	76
38	male	46	T2N1M0	II	1	7

39	male	62	T2N0M0	III	1	4
40	male	49	T2N0M0	II	0	73
41	male	78	T2N1M0	I	0	73
42	male	63	T4N2M0	II	1	64
43	male	48	T3N0M0	II	0	70
44	female	65	T4N2M0	I	0	69
45	male	57	T4N1M0	III	1	57
46	female	58	T2N0M0	II	0	68
47	male	35	T2N0M0	II	0	68
48	male	50	T4N0M0	III	0	67
49	male	48	T2N1M0	II	0	11
50	male	57	T2N0M0	II	0	67
51	male	52	T2N0M0	III	1	21
52	male	59	T2N2M0	II	1	9
53	female	46	T2N0M0	II	1	30
54	male	51	T4N1M0	I	0	65
55	male	61	T1N0M0	II	1	1
56	male	46	T4N1M0	II	0	63
57	male	47	T2N2M0	II	1	3
58	male	63	T4N2M0	II	1	4
59	male	61	T2N0M0	III	1	10
60	male	69	T2N2M0	II	1	17
